# Drug resistance characteristics and risk factors in patients with retreated pulmonary tuberculosis: a 5-year retrospective study in Suzhou, China

**DOI:** 10.3389/fpubh.2026.1748913

**Published:** 2026-04-13

**Authors:** Qinhao Zhou, Yanyang Zhou, Xing Lv, Longji Chen, Dawei Yu, Huafeng Song, Ping Xu

**Affiliations:** 1 Suzhou Medical College of Soochow University, Suzhou, China; 2 The Fifth People’s Hospital of Suzhou, Suzhou, China

**Keywords:** drug susceptibility testing, MTB, pulmonary tuberculosis, risk factor, tuberculosis retreatment

## Abstract

**Objective:**

We conducted a systematic evaluation of TB-infected patient data from January 2020 to December 2024 to statistically analyze retreatment risk factors and drug resistance profiles among culture-positive TB patients in Suzhou.

**Methods:**

This investigation assessed the incidence of TB retreatment and drug resistance patterns in Suzhou, Jiangsu Province, China. Patients were stratified by diagnosis year, sex, age, and disease status. Using individual case records of pulmonary TB patients, drug resistance screening results, and clinical data of drug-resistant patients (01/01/2020–31/12/2024), we employed the chi-square test for group comparisons and multivariate logistic regression to identify influencing factors.

**Results:**

From 2020–2024, a total of 10,898 TB patients were enrolled, including 6,820 *Mycobacterium tuberculosis* culture-positive patients. Among these culture-positive cases, 167 patients required retreatment, yielding an annual incidence of 228 retreatment cases per 10,000 person-years. The incidence of extensively drug-resistant TB (XDR-TB) is 528 per 10,000 person-years. Multivariate logistic regression analysis revealed that anemia, XDR-TB, bronchiectasis, and fatty liver were significantly associated with an increased risk of TB retreatment.

**Conclusion:**

Bronchiectasis, anemia, fatty liver, hepatitis B, and extensively drug-resistant *Mycobacterium tuberculosis* increase the risk of tuberculosis retreatment. Comorbid bronchiectasis is specifically associated with an increased risk of rifampicin resistance, streptomycin resistance, and XDR-TB.

## 1 Introduction

Tuberculosis (TB) remains a global public health concern. According to WHO statistics (1), 10 million people were diagnosed with TB in 2023. Males and females (≥15 years) accounted for 56 and 32% of the cases, respectively, whereas children (<15 years) accounted for approximately 12% of all TB-positive cases that year. Treatment failure after pulmonary tuberculosis (PTB) infection poses a significant challenge—among patients retreated for TB due to initial treatment failure, interruption, or recurrence, drug resistance is common (2). In 2023, 175,923 people with MDR/RR-TB were diagnosed and enrolled on treatment – this accounted for only 44% of the 400,000 estimated incident MDR/RR-TB cases (3). China has estimated 30,000 TB deaths and 30,000 MDR/RR-TB cases, with a downward trend observed since 2015 (4). The prevalence of drug-resistant TB, particularly MDR-TB, threatens China’s economic development and public health, compounded by poor retreatment outcomes. The treatment of TB often requires additional therapy cycles, longer treatment durations, and lower success rates, further facilitating the transmission of *Mycobacterium tuberculosis* strains.

Comorbidities in TB constitute a critical medical and social challenge. Traditional risk factors for TB retreatment (5) include HIV infection, diabetes mellitus, low body weight, cavitation on chest X-ray, high bacterial load, short treatment duration, drug resistance, and positive culture after two months of treatment6. This study conducts a comprehensive analysis of anti-tuberculosis drug resistance and retreatment in Suzhou (2020–2024), aiming to identify risk factors associated with drug resistance and PTB retreatment in Suzhou; predict TB prevention trends based on historical disease progression; summarize the epidemiological characteristics of DR-TB in Suzhou; and identify local patient profiles linked to a high risk of primary TB treatment failure, recurrence, or default.

## 2 Methods

### 2.1 Data sources and ethical statement

All the analyzed data were obtained from The Fifth People’s Hospital of Suzhou. In accordance with national legislation and institutional requirements, written informed consent was not required for participant enrollment in this study. The license period for the dataset used in this study for research purposes is from 01/10/2024 to 01/07/2025, and we completed the data collection and analysis on 15/06/2025.

### 2.2 Study design and population

**Figure 1:**
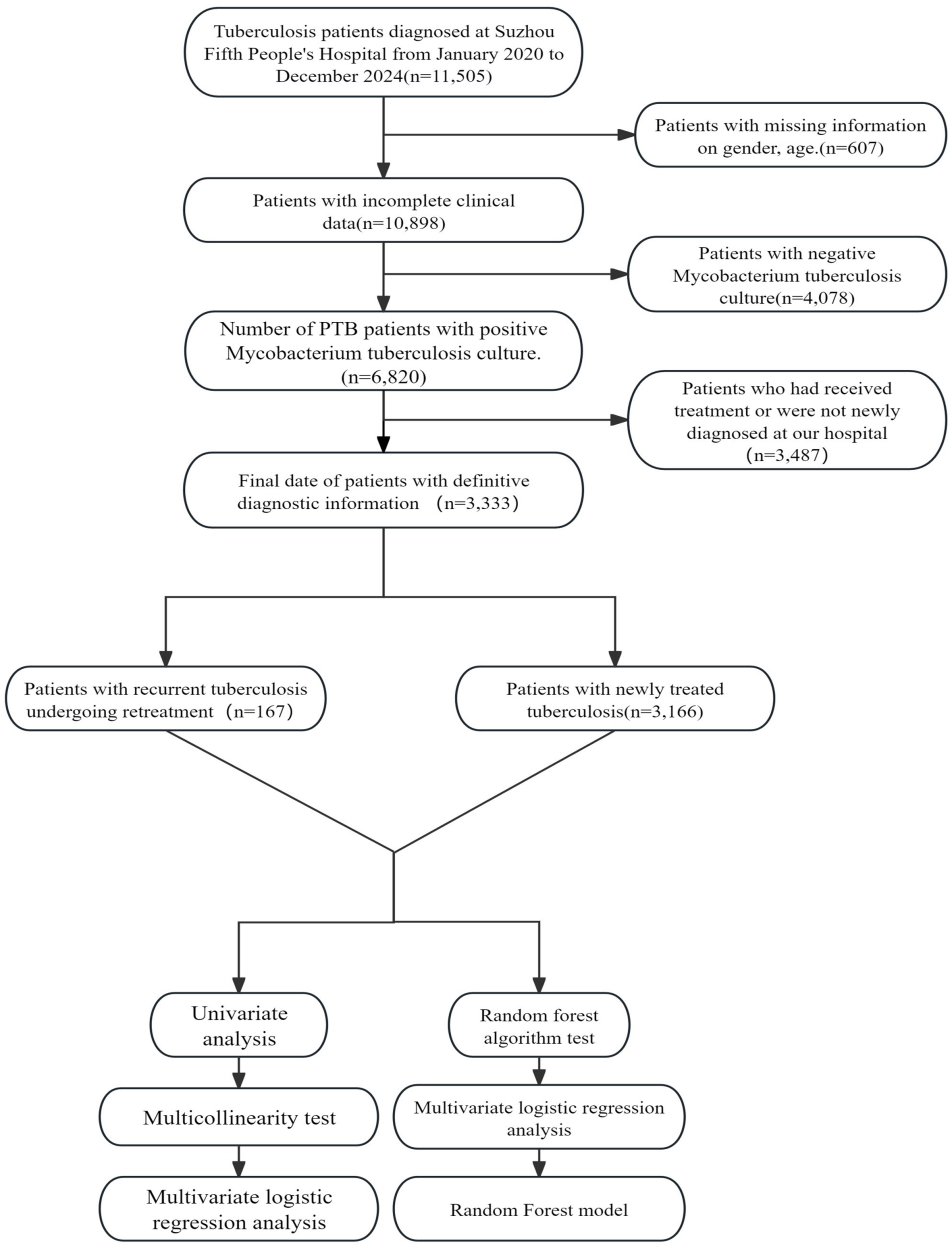
Researchers and statistical flowchart.

This was a retrospective, longitudinal follow-up study of patients with MDR/RR-TB. From January 2020 to December 2024, 11,505 tuberculosis patients were included. After excluding 4,078 culture-negative patients and 607 patients with missing information on sex, age, and disease status, 6,820 patients with *Mycobacterium tuberculosis* culture-positive results were selected finally. Among these 6,820 patients, 3,166 were newly diagnosed with pulmonary tuberculosis at our hospital and initiated standardized treatment, and, 167 were retreated tuberculosis patients. After excluding all patients with incomplete essential information, the remaining patients were classified based on potential risk factors for tuberculosis retreatment, and the outcomes of initial treatment patients were compared with those of retreatment patients (Figure 1).

### 2.3 Relevant definitions

According to the standard definitions in the Guidelines of the US National Tuberculosis and Leprosy Control Program (NLCP), which was adopted by the WHO (6). Retreatment for pulmonary tuberculosis refers to patients with failed initial treatment, patients with positive sputum bacteria after completing a regular treatment course, or patients who had received irregular chemotherapy for more than 1 month. Monoresistance: *Mycobacterium tuberculosis* is resistant to one anti-tuberculosis drug. Multidrug resistance (7): *Mycobacterium tuberculosis* was resistant to at least isoniazid and rifampicin. Extensively Drug-Resistant: On the basis of multidrug resistance, it is resistant to any one fluoroquinolone and any one of the three injectable agents (capreomycin, kanamycin, and amikacin). Rifampicin resistance (6): M tuberculosis is resistant to rifampicin, regardless of resistance to other antituberculosis drugs. Isoniazid resistance: M tuberculosis is resistant to isoniazid, regardless of resistance to other antituberculosis drugs. Extrapulmonary tuberculosis includes tuberculosis in organs other than the lungs, such as the lymph nodes, abdomen, genitourinary tract, skin, joints and bones, and meninges.

### 2.4 Statistical analysis

Data on PTB patients in Suzhou, Jiangsu Province, from 2020.01–2024.12 were obtained from the Laboratory Center of Suzhou Fifth People’s Hospital. Patients with incomplete data (including missing drug susceptibility test results, age, or sex) were excluded to avoid selection bias. Statistical analyses were performed via SPSS version 27.0 (SPSS Inc., Chicago, Illinois). Patient characteristics are described as percentages, medians, ranges, and frequencies. The distribution of drug-resistant TB patients enrolled in treatment was characterized by rates (%). The chi-square test was used to compare categorical variables between groups and identify potential risk factors associated with drug resistance in MDR/RR-TB strains. Basing on the WHO TB diagnosis and treatment guidelines (3), and excluding potential comorbidity factors with a sample size of <40 cases, we initially identified the following variables as candidates for multivariate regression analysis: TB-related characteristics; Clinical comorbidities. Univariate logistic regression was applied to screen for significant risk factors for retreatment, followed by multivariate logistic regression to determine independent risk factors. Variables that showed a p-vale of less than 0.05 in the univariate analysis were included. The results with *p* < 0.05 were considered statistically significant.

**Figure 2:**
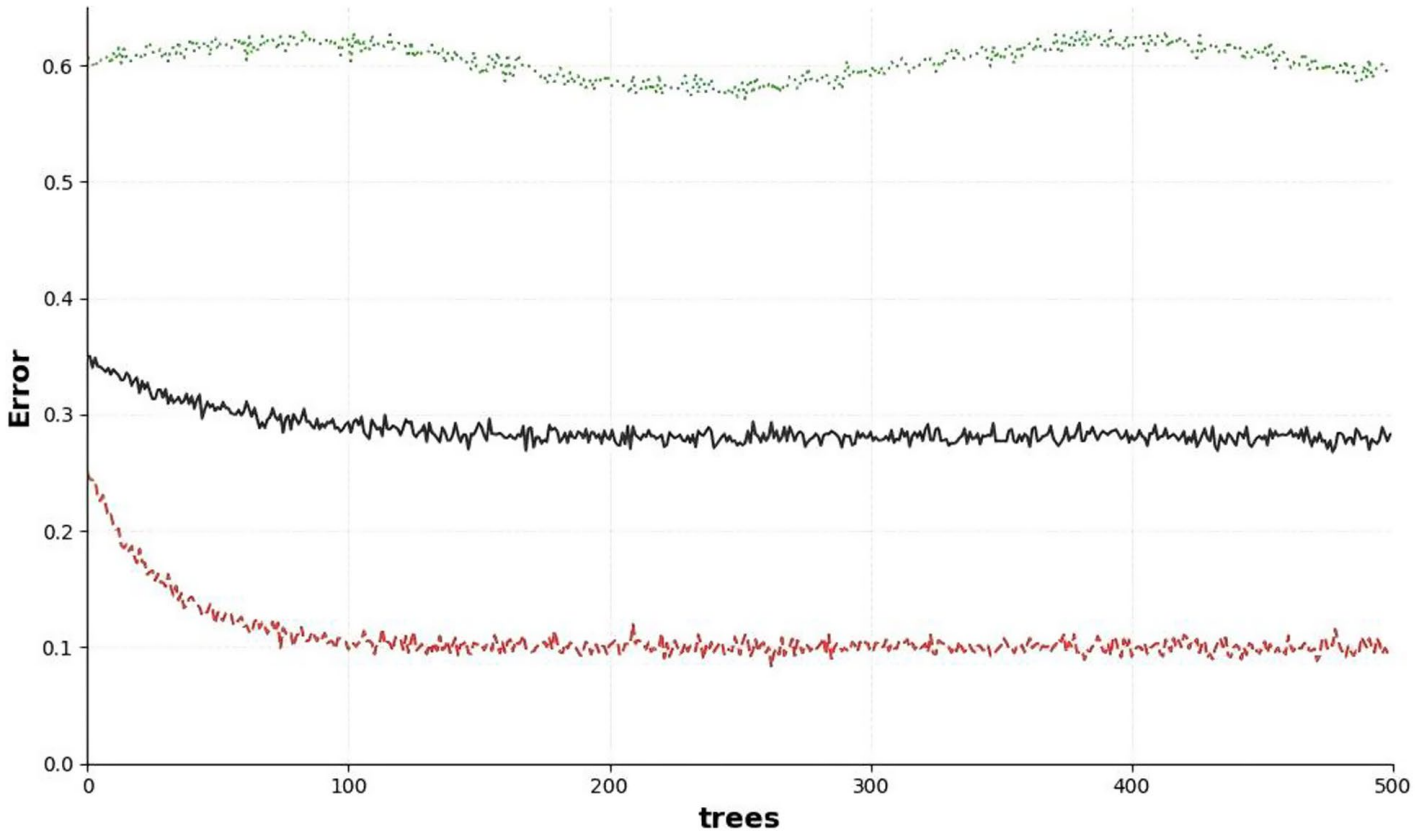
Dynamic relationship between prediction error curve of the random forest model with increasing number of decision trees (ntree).

We used the Random Forest algorithm in R software to build a prediction model, aiming to analyze the relationship between each feature and retreatment status and identify retreatment risk factors. During modeling, the dataset was split into training and test sets at an 80:20 ratio. After training the model on the training set, predictions were generated for both the training and test sets. We calculated evaluation metrics including Precision, Recall, and Root Mean Squared Error. Grid search based on 5-fold cross-validation showed that the model achieved the optimal cross-validation F1 score (0.4562) when mtry = 4. At this point, the accuracy difference between the training set and test set was only 2.14%, indicating no severe overfitting and verifying the rationality of this parameter value. When ntree = 150, the error is basically stable, and the dynamic relationship between the prediction error of random forest and the number of random trees is shown in Figure 2. Precision reflects the reliability of positive retreatment predictions, Recall represents the sensitivity of screening actual retreatment cases, and RMSE quantifies the deviation of predicted retreatment risk from actual outcomes.

### 2.5 *Mycobacterium tuberculosis* drug susceptibility test

Use liquid culture positive samples that have been confirmed as acid-fast bacilli by acid-fast staining, are free from contamination, and are positive for MPB64 antigen. Incubate at 37 °C for 1–7 days. If the bacterial load is insufficient after 7 days, transfer to a new liquid culture tube. Use a sterile pipette to transfer 0.5 mL of bacterial suspension to an ultrasonic dispersion tube containing 1.5 mL of saline. Tighten the cap and sonicate. Adjust the bacterial suspension to 0.5 McFarland using physiological saline as needed. Pipette 100 μL of the bacterial suspension into a 7H9 broth containing OADC. Inoculate the antibiotic susceptibility plate: Use a multichannel pipette or Sensititre Auto Inoculator®/AIM® to add 100 μL of inoculum to each well of the plate. Seal the plate with the provided sealing film, ensuring no wrinkles or gaps. Use a roller or scraper to ensure airtightness. Incubate the sealed plate at 35–37 °C in an aerobic environment for 10 days. Check the growth of control wells between days 7–10. If growth is insufficient after 10 days, extend incubation to day 11.

## 3 Results

### 3.1 Demographic and clinical characteristics of different age ranges

A total of 6,820 culture-positive tuberculosis samples were collected from Suzhou Fifth People’s Hospital. PTB was observed across all age groups (Table 1), with the number of male cases exceeding the number of female cases in each demographic stratum. Among the culture-positive *Mycobacterium tuberculosis* samples, 4,502 (66%) were male, and 2,318 (34%) were female, with ages ranging from 1 to 99 years (mean age: 46 years; median: 44 years).

**Table 1:** Annual distribution of etiologically positive tuberculosis cases and drug resistance detection by gender (2020–2024).

	Male		Female	
Year	Number of etiologically positive cases	Number of drug resistance detected	Number of etiologically positive cases	Number of drug resistance detected
2020	920	72	403	35
2021	992	204	512	150
2022	721	102	371	73
2023	1050	84	564	41
2024	819	104	468	59
Total	4502	566	2318	358

**Figure 3:**
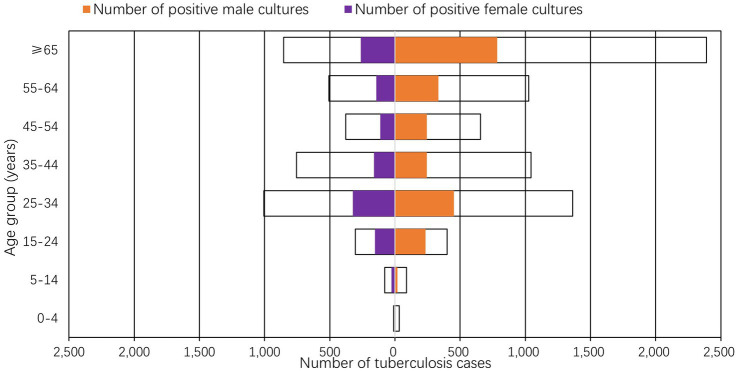
From 2020 to 2024, the number of tuberculosis diagnoses in Suzhou (marked by a black).

Among all culture-positive *M tuberculosis* samples, the male cases included 88 (15–24 years, 1.30%), 400 (25–34 years, 5.86%), 493 (35–44 years, 7.2%), and 493 (45–54 years, 7.2%)—all of which exceeded the female incidence in the corresponding age groups (Figure 3). The age-specific incidence of culture-positive PTB displayed a bimodal distribution. Of 167 retreatment cases, 45.5% were 15–34 years and 32.9% ≥ 65 years, identifying these two age groups as primary retreatment populations.

Across all categories, including rifampicin-resistant tuberculosis, isoniazid-resistant tuberculosis, mono-resistant tuberculosis, MDR-TB, and XDR-TB, male infection rates (ranging from 3.30 to 5.63%) are consistently higher than those of females (ranging from 1.67 to 3.63%) (Table 2).

**Table 2:** Infection rates of different types of tuberculosis infection.

Type	Gender	Sample size	Infection rate (%)
Rifampicin-resistant tuberculosis	Male	317	4.65
	Female	215	3.15
Isoniazid-resistant tuberculosis	Male	225	3.30
	Female	114	1.67
MDR-TB	Male	384	5.63
	Female	248	3.63
XDR-TB	Male	338	4.96
	Female	227	3.32

### 3.2 Characteristics of drug-resistant TB populations

In the total population, the proportions of isoniazid-resistant tuberculosis, XDR-TB, and rifampicin-resistant tuberculosis in males were 10.7, 5.44, and 6.6%, respectively, all of which were higher than the incidence rates of drug-resistant TB in females. A chi-square test revealed significant differences in drug-resistant populations between the initial treatment and retreatment groups (χ^2^ = 44.36, *p* < 0.05), indicating an association between initial/retreatment of PTB and extensive drug resistance. Additionally, patients with comorbid bronchiectasis presented differential TB drug resistance in drug susceptibility testing (χ^2^ = 9.47, *p* < 0.05), suggesting that comorbidities in TB patients may influence drug resistance during treatment.

### 3.3 Univariate and multivariate and random forest model analyses of risk factors for the treatment of secondary pulmonary tuberculosis

Univariate analysis revealed that four factors were associated with a greater risk of TB retreatment and diagnosis. A total of 3,333 newly treated TB patients and 167 retreated TB patients were included as controls to analyze risk factors for retreatment. Univariate analysis (Table 3) revealed seven factors linked to an elevated risk of TB retreatment: MDR, XDR, mono-resistance, bronchiectasis, fatty liver, anemia, and hepatitis B were significantly associated with TB retreatment. However, the current statistical results failed to demonstrate a significant difference in the associations of hepatitis B or bacterial pulmonary infection with TB retreatment (*p* > 0.05).

**Table 3:** The risks of tuberculosis retreatment posed by different types of factors.

Risk factors for retreatment	Number of cases	Univariate logistic regression analysis		Multivariate logistic regression analysis	
		*P*	OR	*P*	OR
Gender					
Male	2,305	0.02	1.44(1.19–1.74)	0.02	1.62(1.03–1.56)
Female	1,184	0.01	——	0.01	——
Tobacco use	1,084	0.01	2.21(1.65–2.97)	0.01	2.14(1.57–2.92)
*Mycobacterium tuberculosis* —resistance type					
Mono-resistance	116	0.21	1.27(0.87–1.85)	——	——
MDR-TB	247	0.01	3.36(1.43–6.89)	0.02	2.84(1.52–5.49)
XDR-TB	208	0.01	4.13(2.64–6.47)	0.01	2.02(1.19–3.42)
Comorbidities					
Anemia	146	0.01	3.27(2.50–4.37)	0.01	4.03(3.04–5.36)
Fatty liver	41	0.01	3.340(1.51–7.37)	0.01	4.07(2.78–5.93)
Bronchiectasis	169	0.04	1.52(1.13–2.05)	0.01	2.32 (1.59–3.37)
Extrapulmonary tuberculosis	834	0.01	0.46(0.30–0.71)	0.34	1.11(0.54–1 0.96)
Hepatitis B	59	0.01	3.48(2.33–5.20)	0.01	4.23(2.65–6.43)
Hypertension	70	0.06	2.76(1.59–4.41)	——	——
Bacterial pulmonary infection	446	0.04	0.56(0.33–0.97)	0.21	0.795(0.56–1.14)

Multivariate logistic regression analysis of the above factors revealed that the risk of retreatment was significantly increased in patients with anemia (OR = 4.03, *p* < 0.05). Patients with XDR had a significantly greater risk of retreatment than nonresistant patients did (OR = 2.02, *p* < 0.05). Bronchiectasis was also a risk factor for TB retreatment and drug resistance (OR = 2.32, *p* < 0.05); Bronchiectasis was also a risk factor for TB retreatment and drug resistance (OR = 2.32, *p* < 0.05); patients with bronchiectasis were more prone to developing XDR-TB than those without bronchiectasis, with a correlation between the higher risk of XDR-TB in bronchiectasis patients and an elevated retreatment risk. The risk of retreatment was significantly increased in patients with fatty liver (OR = 4.07, *p* < 0.05). In contrast, extrapulmonary *tuberculosis* had no significant impact (OR = 1.11, *p* > 0.05). In the multivariate logistic regression analysis, the Hosmer–Leme show test showed a good fit (*p* = 0.49 > 0.05, χ^2^ = 6.45). The multivariate logistic regression results (Table 3) revealed that all the independent variables had variance inflation factors (VIFs) between 1.009 (monoresistance) and 4.321 (multidrug resistance), with all values < 5. This indicated no significant multicollinearity, supporting the stability, reliability, and credibility of the regression findings.

**Figure 4:**
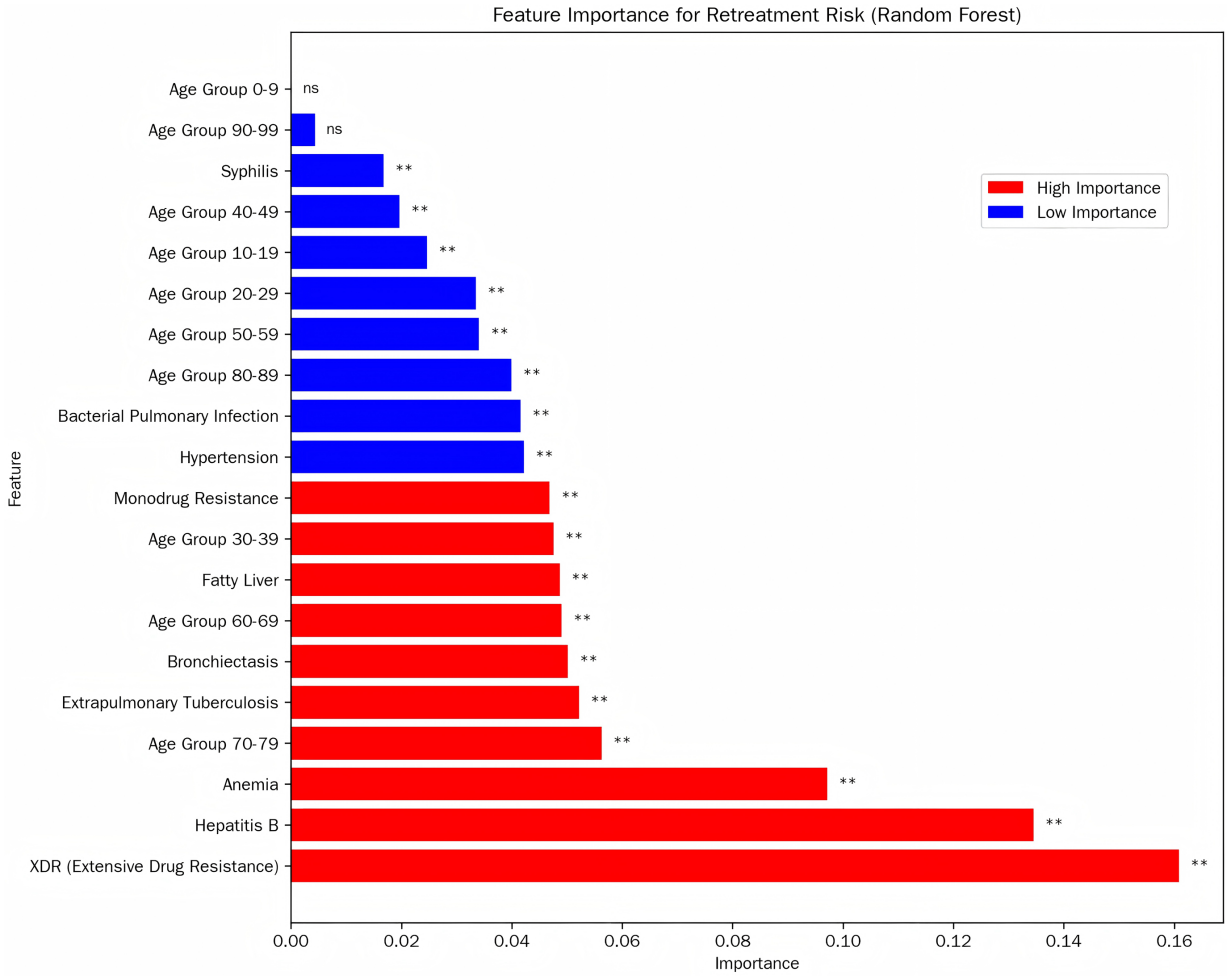
Random forest feature of different types of factors.

According to the results (Figure 4), XDR, HBV, Anemia, age group 70-79, Extrapulmonary Tuberculosis, Age group 60-69, Fatty Liver, Age group 30-39, Mono-durg Resisance were the top ten critical factors for predicting depression in PTB patients. Demonstrates the ranking of importance of these indicators.

## 4 Discussion

Drug-resistant tuberculosis (DR-TB) and tuberculosis retreatment have become increasingly severe public health challenges in China. Our study revealed that the incidence of XDR-TB in Suzhou was 528 per 10,000 person-years. Compared with the global proportion of 3.3% XDR-TB among new patients in 2022, the incidence of XDR-TB in Suzhou is relatively high and shows an increasing trend (8). Among the total population, male patients had significantly higher prevalence rates of isoniazid resistance (10.7%), extensive drug resistance (5.44%), and rifampicin resistance (6.6%) than female patients did, which is consistent with the global trend of a greater tuberculosis burden in males ^4^. This sex difference may be related to greater exposure risks in males (e.g., occupational environments, smoking habits) or differences in immune responses (9). In children, we found a significantly lower prevalence in those under 15 years of age, which may be associated with the positive control effect of the neonatal BCG vaccination program. A 14-year cross-sectional study on tuberculosis in China revealed that the risk in the 15–34 age group in eastern China was significantly higher than that in other regions (10, 11), which supports the observation that tuberculosis patients in Suzhou are mainly concentrated in the 15–34 and over 65 age groups, showing a bimodal trend. The high prevalence of tuberculosis in young people may be mainly associated with ongoing transmission within communities, such as aggregation in schools and frequent social activities, which increase exposure risks. For elderly individuals over 65 years of age, the high prevalence of tuberculosis is associated with weakened immunity and comorbid chronic diseases.

The incidence of retreatment tuberculosis in Suzhou (228 per 10,000 person-years) falls within the range of incidence rates reported in low-incidence areas. A study in England and Wales from 1998--2006 revealed that the recurrence rate of culture-positive tuberculosis patients was 660 per 10,000. In Suzhou, the retreatment rate among culture-positive retreatment of tuberculosis patients was 2.3% (167/6820), whereas in Shanghai, a total of 5.3% (710/13,417) of successfully treated patients underwent retreatment (5); thus, the retreatment rate in Suzhou (2.3%) was lower than that in Shanghai (5.3%). There was a significant difference in the XDR population between initially treated and retreated patients, suggesting that drug resistance may be exacerbated during retreatment (12), which is closely related to poor treatment adherence or irregular initial treatment regimens.

In this study, analysis of the tuberculosis population in Suzhou revealed that patients with pulmonary tuberculosis complicated by bronchiectasis had a 2.32-fold increased risk of retreatment. Bronchiectasis may be associated with changes in the lung tissue structure, making it easier for bacteria to spread (13); moreover, impaired mucus clearance promotes the colonization and reproduction of *M tuberculosis* (14). In addition (15), bronchiectasis itself may hinder the complete delivery of antibacterial drugs to the infection site, thereby reducing treatment efficacy and potentially gradually affecting patients’ lung function (16). Patients with fatty liver had a 4.07-fold increased risk of retreatment. Fatty liver is associated with increased retreatment risk, potentially via the following pathways: patients with fatty liver may face a greater risk of drug-induced liver injury (DILI) when receiving tuberculosis treatment, and the treatment effect may be affected (17). Liver damage leads to abnormal metabolism of anti-tuberculosis drugs, increasing the risk of treatment interruption; fatty liver-related inflammatory responses may interfere with immune system function, reducing the body’s ability to clear *Mycobacterium tuberculosis* (18); and the treatment course for patients with fatty liver and liver damage is prolonged owing to hepatoprotective therapy, and longer courses increase the probability of missed doses or treatment interruption (19). Anemia may increase the short-term risk of tuberculosis recurrence and retreatment by 4.03-fold. Previous studies have widely suggested that malnutrition may negatively affect treatment outcomes (20); malnutrition and anemia are common in patients with tuberculous meningitis, especially those with concurrent PTB and Nontuberculous Mycobacteria (NTM), and anemia causes short-term immune decline in patients. Notably, the retreatment population shows two age peaks: 15–34 years and over 65 years. The former, as the primary labor force, may undergo retreatment due to high work pressure and high treatment interruption rates; the latter is associated with age-related immune decline and increased comorbidities. The Random Forest model with good stability further ranked retreatment risk factors, and its Precision, Recall and RMSE ensured the model’s clinical applicability—sensitively screening high-risk retreatment patients and reliably stratifying clinical risk for targeted intervention.

Finally, the results of this study are consistent with those of the WHO report, indicating that the proportion of cases among retreated patients in China is 13% (1). Still, we identified a specific association of XDR-TB with the retreatment population, supplementing regional data on drug resistance. In addition, the significant association between fatty liver and retreatment found in the Suzhou population, unlike traditional factors such as HIV and diabetes, may be related to the high incidence of metabolic syndrome in the Yangtze River Delta region. The study also revealed that comorbidities such as diabetes and anemia in young people closely associated with retreatment, which is consistent with the current situation of high social pressure and insufficient management of chronic diseases in this group.

However, this study has several limitations: As a retrospective study, there may be residual confounding factors; The data were exclusively derived from the Fifth People’s Hospital of Suzhou, a regional tertiary referral center for tuberculosis in Jiangsu Province, limiting generalizability; the indicators in this study are limited to existing variables, which may introduce selection bias. Socioeconomic factors such as smoking and alcohol abuse are closely associated with the development of MDR/RR-TB and poor treatment outcomes. However, these confounding factors were excluded in this study to avoid the influence of social factors on the model of comorbid tuberculosis. Future prospective studies involving multiple medical centers should expand the research variables for further verification. This study concludes that due to the detection of the progressive transmission of tuberculosis in the population, especially in the working-age group, Suzhou should take action to avoid the increasing impact of tuberculosis on national health and strengthen publicity targeting populations at risk of tuberculosis. These findings inform targeted TB prevention in Suzhou, prioritizing interventions for young working-age and older population with comorbidities to reduce retreatment and drug-resistant transmission.

## Data Availability

The original contributions presented in the study are included in the article/supplementary material, further inquiries can be directed to the corresponding author/s.
